# Is the Rotatory Knee Stability Immediately Decreased Following a Competitive Soccer Match?

**DOI:** 10.3389/fbioe.2022.903131

**Published:** 2022-07-22

**Authors:** Alejandro Neira, Rony Silvestre, Aníbal Debandi, Daniel Darras, Iver Cristi-Sánchez, Ignacio Barra, Luis Peñailillo, Carlos De La Fuente

**Affiliations:** ^1^ Escuela Kinesiología, Facultad de Ciencias, Universidad Mayor, Santiago, Chile; ^2^ Unidad de Biomecánica, Centro de Innovación, Clínica MEDS, Santiago, Chile; ^3^ Carrera de Kinesiología, Departamento de Cs. de la Salud, Facultad de Medicina, Pontificia Universidad Católica de Chile, Santiago, Chile; ^4^ Traumatología, Clínica MEDS, Santiago, Chile; ^5^ Servicio Traumatologéa, Hospital Clénico Universidad de Chile, Santiago, Chile; ^6^ Laboratorio de Neuromecánica Aplicada, Escuela de Kinesiología, Facultad de Ciencias Médicas, Universidad de Santiago de Chile, Santiago, Chile; ^7^ Exercise and Rehabilitation Sciences Laboratory, School of Physical Therapy, Faculty of Rehabilitation Sciences, Universidad Andres Bello, Santiago, Chile; ^8^ Laboratory of Neuromechanics, Universidade Federal do Pampa, Campus Uruguaiana, Uruguaiana, Brazil

**Keywords:** anterior cruciate ligament, sports medicine, soccer (football), re-rupture, risk factors

## Abstract

Fatigue induced by soccer playing increases physical efforts, which might alter the transverse knee stability, a known factor that promotes knee injuries, particularly anterior cruciate ligament injury. Thereby, primarily, we aimed to determine whether rotatory knee stability decreases immediately following a competitive soccer match in amateur players. Furthermore, we assessed the role of the preferred and non-preferred limbs to kick a ball in rotatory knee stability and the correlation between performance parameters and rotatory knee stability. We hypothesized that the knee stability decreases immediately after a competitive soccer match in amateur players. Eight healthy amateur soccer players (aged 27.2 ± 4.7 years and with body mass index of 23.8 ± 1.2 kg m^−2^) were included immediately before and after a competitive soccer match. The rotatory knee stability was assessed in the preferred and non-preferred limbs through the acceleration and jerk of the pivot shift maneuver and by the internal knee rotation of a pivoting landing task. Two-way repeated-measures ANOVA for factors time (before and after the soccer match) and limb (preferred and non-preferred) and multiple comparisons were performed using *α* = 5%. There was a statistical significance for the main factor time in the acceleration (5.04 vs. 6.90 ms^−2^, Δ = 1.86 ms^−2^, *p* = 0.020, η^2^ = 0.331) and jerk (18.46 vs. 32.10 ms^−2^, Δ = 13.64 ms^−2^, *p* = 0.004, η^2^ = 0.456) of the pivot shift maneuver. Rotatory stability decreases following a competitive soccer match in amateur soccer players under fatigue. Both the acceleration and jerk during the pivot shift maneuver is increased without significant internal knee rotation changes during the pivoting landing task.

## Introduction

Soccer playing is a prolonged high-intensity intermittent exercise that lasts ∼90 min in professional activity (Orendurff et al., 2010). Players repetitively make forays across the anaerobic threshold followed by recovery epochs (Orendurff et al., 2010). In contrast, prolonged exercise is associated with acute kinematics changes that impair at least the landing tasks (Shultz et al., 2015). Both strength and activation loss, and kinematic pattern changes under muscle fatigue are characterized by loss of a degree of freedom to protect against muscle damage (Baumert et al., 2021). In addition, repeated eccentric contractions such as those that occur in soccer matches increase muscle damage in knee muscles (Baumert et al., 2021). Also, a delayed recovery with a lack of kinematic control can occur for even more than 48 h post-exercise (Baumert et al., 2021). On the other hand, intermittent exercises simulating the intensity and duration of a soccer match has been associated with increased internal knee rotation compared with rest and no fatigued conditions (Shultz et al., 2015). Thus, it appears that the fatigue induced by soccer playing can decrease rotatory knee stability, allowing uncontrolled pivoting movements of the knee (Shultz et al., 2015).

The fatigue induced by soccer playing impairs several cognitive and tactical strategies, increasing tactical errors and physical efforts (Kunrath et al., 2020). These increased efforts are associated with peripheral fatigue (Carroll et al., 2017), which reduces the sensorimotor control and proprioception during a match (Robineau et al., 2012). Evidence suggests that at least 70% of anterior cruciate ligament (ACL) will be ruptured as a non-contact mechanism (Alentorn-Geli et al., 2009), with emphasis on amateur athletes (Montalvo et al., 2019), in previous ACL ruptures (Alentorn-Geli et al., 2009), and mainly during the second half of the match where the fatigue induced by soccer playing is more evident (Faunø and Wulff Jakobsen, 2006). However, as the force restitution can last at least 30 min after a match (Carroll et al., 2017), immediate measurements should be developed to achieve conclusions on uncontrolled knee pivoting induced by soccer playing closer to a real competition. In addition, researchers have indicated that most fatigue protocols cannot reflect lower limb fatigue such as what happens in soccer matches, also limiting the outcomes (Hantes et al., 2012).

On the other hand, the rotational knee stability allows an appropriate tibio-femoral displacements and knee axis shifting into physiological ranges (Bull and Amis, 1998). The pivoting knee movements are expected to be controlled by knee co-contraction (Caracciolo et al., 2021) and by an adequate knee laxity (Bull and Amis, 1998). In general, the rotational knee stability is measured by the pivot shift maneuver that elicits the coupled movement between the anterior displacement and medial rotation of the tibia while a knee flexion is developed. When the ACL is insufficient, the lateral tibial plateau displaces more anteriorly and medially (subluxation), and because the iliotibial band is stretched, a sudden higher posterior and lateral shift of the lateral tibial plateau (reduction) is caused (Caracciolo et al., 2021). In support, the pivoting landing task has been implemented to explore the excessive internal knee rotation in a closed kinetic chain (excessive knee rotation), increasing the rotational load on the knee joint (Pappas et al., 2013). However, this last testing could develop a higher coefficient of variation ranging between 26 and 35% (Ristanis et al., 2003).

The fatigue protocol modality and measuring methods are crucial to assess knee stability, i.e., instrumented pivot shift maneuver and 3D pivoting landing task (Ristanis et al., 2003). Moreover, a real competitive soccer match has been recommended as a good model for prolonged high-intensity intermittent fatigue. But without immediate measurements, no appropriate conclusions on knee pivoting might be achieved. Therefore, primarily, we aimed to determine whether rotatory knee stability decreases immediately following a competitive soccer match in amateur players. Furthermore, we assessed the role of the preferred and non-preferred limbs to kick a ball in rotatory knee stability as well as the correlation between performance parameters and rotatory knee stability. We hypothesized that knee stability immediately decreases after a competitive soccer match in amateur players.

## Materials and Methods

### Study Design

In this cross-sectional, analytical, and observational study, we measured the rotatory knee stability in competitive amateur soccer players who used their preferred and non-preferred limbs to kick a ball before and immediately after a competitive (80 min) soccer match motivated to win the season. We set a biomechanical laboratory close to the soccer field starting 8 h before the assessments to achieve immediate measurements (<30 min per person; Table 1). The soccer match was played in the summer season. The weather is typically dry and has an average temperature of 74.8°F (23.8°C), average humidity of 42%, average daylight of 14.3 h, and average sunshine 9.3 h. The grass field was 76.6 yards (70 m) wide and 98.4 yards (90 m) long. The match was performed at 570 m above sea level, with clear weather, at 8 p.m., with ∼64.4°F (18°C) temperature, ∼25% humidity, and ∼1,011 mbar pressure. All participants gave their written informed consent prior to participating in the study. The local ethical committee approved this study with code #25030219 (Santiago, Chile) and according to the principles of the Helsinki Declaration.

**TABLE 1:** Soccer match demands and time of kinematic assessment.

	Mean ± SD / Median [min max]
Total distance covered, *m*	7,454.5 ± 784.6
Number of efforts at high intensities (>18 km/h), *No.*	26.8 ± 15.1
Average heart rate, *bpm*	164.5 ± 11.3
Distance at high-intensity speed (>18 km/h), *m*	707.8 ± 377.2
Maximum speed reached, m*s ^*−1*^	7.4 ± 0.5
Physical load (player load), *UA*	751.0 ± 118.9
Time of kinematic assessment, *min per person*	4.0 ± 2.1
Borg perception, *pts*	7 [7–8]

SD, standard deviation; m, meters; No., number; bpm, beats per second; s, seconds; min, minimal; max, maximal.

UA, arbitrary units (Barrett et al., 2014).

### Participants

Eight healthy amateur soccer players (age: 27.2 ± 4.7 years; height: 178.0 ± 0.1 cm; body mass: 75.5 ± 7.3 kg; body mass index: 23.8 ± 1.2 kg m^−2^; and 9.5 ± 2.0 years playing soccer continuously) were enrolled in this study. The players reported an average level of physical activity of 12.0 ± 0.9 pts according to the Marx scale, which focuses on four activity points: running, deceleration, cutting (changing directions while running), and pivoting (Shultz et al., 2015). The preferred limb to kick a ball was defined as one that the player uses to kick the ball with maximal dexterity (Bezuglov et al., 2020). In this study, kicking a ball is understood as the multiarticular movement characterized by a proximal-to-distal motion of the lower limb segments of the kicking leg where the angular velocity is maximized first by the thigh, then by the shank, and finally by the foot. Segmental and joint movements follow this neuromechanical strategy in multiple planes (Kellis and Katis, 2007).

The total estimated sample size of the study was seven participants using an *α* and *β* set to 0.05 and 0.10 (statistical power [1 − *β*] of 0.90, which typically ranges between 0.05 and 0.20) (Banerjee et al., 2009), respectively. The effect size (E.S.) was obtained from a within-group difference of 4.02° (large E.S. of 1.4) in tibial rotation from a previous study (Giotis et al., 2013). We set the dropout of the study to 35%, aiming to recruit 11 soccer players. Three players decided not to participate in the last measurements by personal assumptions because they lost the final game. Thus, the final sample was eight participants instead of 11.

To be included in this study, all participants must play regularly in an amateur soccer league each weekend during 40 weekends divided similarly into two seasons and be part of the same team. The exclusion criteria of the study are as follows: those who do not have previous medical reports of knee injuries and any injury at the moment of the measurements, those who played for more than 60 min, those who have consumed alcohol or have taken nonmedically prescribed drugs in the week prior to the match, those who have completed all assessments, and those with a Borg scale rating lower than 5 (hard perception of the effort) (Foster et al., 2001). However, no patients were excluded by values lower than 5.

### Performance Parameters

The physical demands (performance parameters) of the soccer match were characterized through a wireless monitoring system (Optimeye S5, Catapult Sports, Australia) with a sampling frequency of 10 Hz. The physical performance measurements included the average heart rate (bpm), distance (m), average speed (km/h), and the player load (workload) (Barrett et al., 2014). The physical performance of players was analyzed using the software Sprint 5.0 (Catapult Sport, Australia). Also, the modified rate of perceived effort was measured before and after the match using the Borg scale (range of 1–10 pts, where 0 indicates no physical effort sensation and 10 indicates the maximal physical effort sensation) (Wilson and Jones, 1989).

### Testing Protocol

We performed the pivot shift maneuver using accelerometry and the dynamic pivoting landing task using 3D-kinematics to evaluate the rotatory knee stability. Prior to the soccer match, participants performed 10 min of familiarization, consisting of 10–15 trials for each measurement. Verbal feedback was provided to correct the performance in each test. Once this period was concluded, the data acquisition of the pre-match condition was collected. First, the pivot shift maneuver was repeated three times for both the preferred and non-preferred limbs to kick a ball. Afterward, data from three successful pivoting landing tests on each limb were collected. Then, participants performed a competitive (80 min) soccer match (fatigue induced by soccer playing). Thereafter, the match, the knee stability was again assessed. In conclusion, the lasting time for the post-match condition assessment was registered.

### Instrumented Pivot Shift Maneuver

A senior surgeon developed the instrumented pivot shift maneuver while the patient lay down in a supine position on a table, according to Musahl et al. (2012). The movement was tracked using a triaxial accelerometer (Myomonitor^®^ IV wireless system, Delsys Inc., United States). The sample rate was 10 kHz. The accelerometer was placed in the middle point between the patient’s Gerdy’s tubercle and the tibial tuberosity with the patient in a supine position and was aligned with respect to the longitudinal axis of the tibia bone (Abusleme et al., 2021). The final position was marked using a permanent blue marker for reproducibility in the pre-match and post-match conditions. The test was repeated three times, obtaining the mean of both the preferred and non-preferred limbs to kick a ball. The delta between the maximum and minimum from the modulus of the acceleration during the reduction phase of the pivot shift maneuver was used (Caracciolo et al., 2021). The modulus of the acceleration has been estimated from the Euclidean norm 2 as $a_{2}=\sqrt[{2}]{a_{x}^{2}+a_{y}^{2}+a_{z}^{2}}$ in m*s^−2^. We chose the delta acceleration of the resultant acceleration to obtain a physical measure that can transduce equally the flexion, valgus, and internal rotation induced by the clinical test on the patient’s knee, where more acceleration refers to a higher shifting of the axial rotatory axis of the knee (Caracciolo et al., 2021).

Also, the jerk during the reduction phase of the pivot shift test was estimated as the first derivative from the modulus of the acceleration (Pan et al., 2020). Jerk is a higher derivative that involves the rate of acceleration. In this study, the rate of accelerometry represented the smoothness degree of the shifting acceleration of the axial rotatory axis of the knee. The reliability of a jerk in the knee was found between 0.70 for the control limb and 0.80 for the injured knee (Lopomo et al., 2012), while the slope of accelerometry (jerk) has correctly identified the injured knee between 74 and 76% of patients (Napier et al., 2021).

### Pivoting Landing Task

A biomechanical laboratory setup was located close to the soccer field in a room with appropriate sunlight protection to assess the internal knee rotation using six infrared cameras (Motion Analysis Digital Hawk, United States) sampled at 200 frames per second. According to the Helen Hayes model, 19 spherical and reflective markers were placed on bony landmarks of the lower limbs and pelvis (Kadaba et al., 1990). Prior to the testing, a static trial was performed for skeletal normalization purposes and to obtain an adequate alignment (neutral axis of knee rotation) for each participant. The markers were re-aligned whenever the evaluator saw a non-physiological movement in the frontal and transverse planes during a simple flexion–extension movement. He relocated the knee markers until the most physiological location was acquired. The final location of markers was drawn using a permanent blue marker to ensure the reproducibility of the first assessment.

**FIGURE 1:**
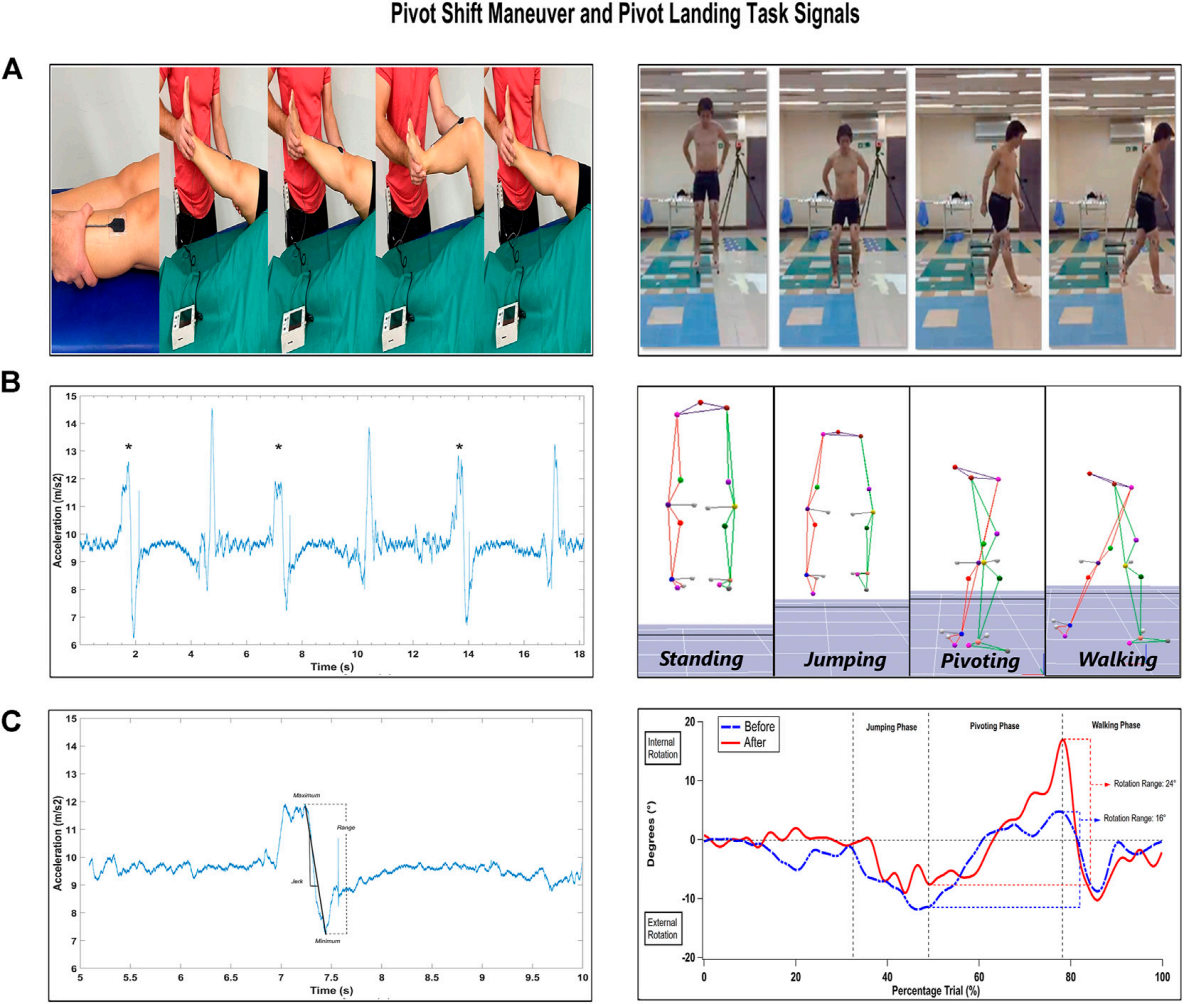
Knee stability measurement: the pivot shift maneuver (left column) and pivot landing task (right column). **(A)** the sequences of the biomechanical assessments. **(B)** the acceleration modulus time series and the reconstructed tridimensional kinematic model. **(C)** the assessment outputs (acceleration range and jerk, and internal rotation range). The pivot landing task shows the standing phase, which consists of the participant standing and waiting for the order to descend. The jumping phase is the task when the participant experiences push-off, flight, and landing. The pivoting phase is the task when the subject lands and turns toward the lateral side. In conclusion, the walking phase is the moment when the participant walks in a horizontal straight line.

Prior to and after the soccer match, the participants were asked to jump from a 40-cm-high platform, land with both feet, and immediately rotate 90° in the clockwise direction, maintaining the ipsilateral foot in contact with the ground. At the same time, the contralateral foot oscillates in front, causing an internal knee rotation in a closed chain (Bohn et al., 2015) (Figure 1). The limb that starts the task was simply randomly assigned at the measurement moment. The participants performed four trials for familiarization with the protocol, and then three successful tests on each limb were recorded. The kinematic marker of the calcaneus was used to identify the different phases of the task.

The kinematic trajectories were gap-filled using cubic functions that were reconstructed and checked by two independent researchers when necessary using the Cortex software (Motion Analysis, inc., United States) (Figure 1). Once reconstructed, the data were low-pass filtered using a second-order Butterworth digital filter with a cut-off frequency of 6 Hz. Then, the inverse kinematic was obtained using the Orthotrack software 6.61 (Motion Analysis, United States). The time series of the jump tasks were analyzed in the pivot phase, where the internal rotation of the knee is developed while the body rotates in the clockwise direction over the foot in contact with the ground. The femoral–tibial rotation range values were normalized to the neutral position of the knee obtained from the static trial.

In conclusion, the angular range of motion was estimated as the delta between the maximum and minimum tibial rotation from the transverse plane during the pivot phase of the jump (Ristanis et al., 2003) (Figure 1).

### Statistical Analysis

The pivot shift acceleration and jerk and the internal rotation of the jumping test are presented as mean and standard deviation. The normal distribution of the data was checked using the Shapiro–Wilk test and Levene’s test (*α* = 5%). The continuous variables were compared using a two-way repeated-measures ANOVA for factors time (before and after the soccer match) and limb (preferred and non-preferred limbs to kick a ball) for pivot shift acceleration and jerk, and internal rotation with *α* = 5%. Sphericity was checked using Mauchly’s test (*α* = 5%). Multiple comparisons were performed with *α* = 5%. The partial eta-square (ŋ^2^) and E.S. of pair comparisons were described. The correlation between performance parameters and knee stability was analyzed using the Pearson correlation coefficient. All statistical analyses were conducted using the SPSS 20.0 software (IBM Inc., United States).

## Results

The soccer match demands (performance parameters) and the time of execution for the measurements obtained in the study are summarized in Table 1. The results of the study are summarized in Table 2, and the correlation between performance parameters and knee stability in Table 3. There was no statistical significance for the main factor limb (*p* = 0.277, ŋ^2^= 0.084) and for the time factor (*p* = 0.915, E.S. = 0.001) in the internal knee rotation. Also, there was no statistical significance for the main factor limb in the acceleration (*p* = 0.198, ŋ^2^= 0.116) and jerk (*p* = 0.338, E.S. = 0.066) of the pivot shift maneuver. However, there was statistical significance for the main factor time in the acceleration (5.04 vs. 6.90 ms^−2^, Δ = 1.86 ms^−2^, *p* = 0.020, ŋ^2^ = 0.331) and in the jerk of the pivot shift maneuver (18.46 vs. 32.10 ms^−2^, Δ = 13.64 ms^−2^, *p* = 0.004, ŋ^2^= 0.456). The acceleration of the pivot shift maneuver after the soccer match in the preferred limb did not show differences with respect to the values prior to the soccer match (5.71 vs. 7.00 ms^−2^, Δ = 1.29 ms^−2^, *p* = 0.080, E.S. = 0.60). The acceleration of the pivot shift maneuver after the soccer match in the non-preferred limb was higher than that prior to the soccer match (4.37 vs. 6.80 ms^−2^, Δ = 2.43 ms^−2^, *p* = 0.013, E.S. = 1.75). The jerk of the pivot shift maneuver after the soccer match in both the preferred limb (19.95 vs. 33.82 ms^−2^, Δ = 13.87 ms^−2^, *p* = 0.013, E.S. = 1.15) and the non-preferred limb (16.97 vs. 30.37 ms^−2^, Δ = 13.40 ms^−2^, *p* = 0.003, E.S. = 1.65) was higher than that prior to the soccer match.

**TABLE 2:** Results of the study.

	Before the match		After the match	
	Preferred	Non-preferred	Preferred	Non-preferred
	*mean ± SD*	*mean ± SD*	Mean ± SD	Mean ± SD
Internal Knee Rotation,*°*	14.14 ± 4.45	16.83 ± 7.03	15.39 ± 4.06	15.08 ± 3.96
Acceleration of PSM, *ms* ^*−2*^	5.71 ± 2.58	4.37 ± 1.62^**Ψ**^	7.00 ± 1.65	6.80 ± 1.10Ψ
Jerk of PSM, *ms* ^*−3*^	19.95 ± 9.63^**ƺ**^	16.97 ± 6.77^**ɣ**^	33.82 ± 14.08 ƺ	30.37 ± 9.25ɣ

SD, standard deviation; m, meters; s, seconds; PSM, pivot shift maneuver.

^Ψ:^ Statistical significance for the non-preferred limb comparing before and after the match (*p* < 0.05).

^**ƺ:**^ Statistical significance for the preferred limb comparing before and after the match (*p* < 0.05).

^**ɣ:**^ Statistical significance for the non-preferred limb comparing before and after the match (*p* < 0.05).

**TABLE 3:** Correlation matrix between performance parameters and knee stability.

	Preferred limb			Non-preferred limb		
	Internal knee rot.	Accel. of PSM	Jerk of PSM	Internal knee rot.	Accel. of PSM	Jerk of PSM
Total distance covered	−0.02	0.03	−0.44	0.03	−0.64	−0.64
No. of efforts at high intensities	−0.50	−0.13	−0.13	−0.35	−0.44	−0.60
Average heart rate	−**0.64**	0.00	−0.57	−0.59	−0.53	−0.57
Distance at high-intensity speed	−**0.75**	−0.46	−0.01	−0.92	−0.25	−0.25
Maximum speed reached	−0.58	−0.21	0.08	−0.66	−0.02	0.11
Physical load (player load)	−0.07	0.17	0.32	0.04	0.42	0.56

No., number; rot., rotation; accel., cceleration; PSM, pivot shift maneuver. Moderate (0.60–0.79) to very high (0.80–1.00) correlations are in bold.

No statistical significance was found (*p* < 0.05).

## Discussion

The most important finding of our study that the rotatory stability decreases immediately after a competitive soccer match in amateur soccer players. Amateur soccer players have shown increased acceleration and jerk during the pivot shift maneuver without significant internal knee rotation during the pivoting landing task. The pivot shift maneuver produces a large peak of anterior tibial translation followed by a reduction phase (acceleration of posterior translation) (Kitamura et al., 2013), shifting the knee rotation axis (Bull and Amis, 1998). Our findings suggest that the workload induced by soccer playing can decrease rotatory knee stability, allowing uncontrolled shifting of the rotation knee axis. This finding agrees with the biomechanical behavior found for deficient ACL knees (Lam et al., 2011) and rotatory instabilities identified by the pivot shift maneuver (Caracciolo et al., 2021). In contrast, the lack of rotatory knee stability is an important risk factor for non-contact ACL rupture (Quatman et al., 2010). Thus, an increased overload risk for meniscus and articular knee surfaces can end in knee osteoarthritis (Webster and Feller, 2019). Regarding the limb preference to kick a ball in soccer, although the non-preferred limb is responsible for stabilizing (greater electromyography activity of the gluteus medius, gluteus maximus, hamstrings, vastus lateralis and medialis, and gastrocnemius) the body in the space during the biomechanics of side-foot-kicking (Kellis and Katis, 2007) and because half of the injuries occur in the limb that kicks the ball (Brophy et al., 2007), we did not identify differences between limb preferences after a competitive soccer match.

Our results showed that soccer players were under fatigue because they provided an abbreviated Borg scale in accordance with a fatigue status of 7/10 pts (“very, very intense activity”) (Wilson and Jones, 1989; Foster et al., 2001). In our study, we have used the rate perception effort modified Borg scale [0 to 10], which is a psychophysiological indicator of fatigue in soccer players (Banerjee et al., 2009; Polito et al., 2017). In addition, this instrument is the most used instrument to measure exertion and fatigue and has a correlation with the training load of soccer players (Polito et al., 2017). The players of our study covered a distance ∼68%–∼79% (from 9,400 to 10,900 m) that is covered in professional soccer.(Russell et al., 2015). Therefore, it is possible to support that high workload (fatigue) induced by soccer playing may have influenced the rotational knee testing observed in our study after a competitive soccer match. In contrast, when fatigue is established, soccer players develop higher physical efforts since players focus on tactical aims without controlling the physical overload (Kunrath et al., 2020). Previous experiments in competitive soccer players who served as a control group after participating in 1 h of soccer-specific field training sessions have found that fatigue can compromise the quality of knee movement (van Melick et al., 2019). In addition, our findings are in accordance with the kinematic alterations showed by soccer players 72 h after have played a soccer match due to the muscle damage and physical well-being is not fully restored (Silva et al., 2018). Thus, the health care team should carefully consider this acute motor adaptation during the match, and the surveillance of the fatigue during the season should be an aim for better management of the musculoskeletal injuries.

In our study, the pivot shift acceleration for the preferred limb and the jerk for the preferred and non-preferred limbs were higher after a competitive soccer match. The acceleration of the pivot shift indicates an altered knee joint rotation (subluxation phenomena) (Caracciolo et al., 2021). At the same time, the jerk measurement is a higher-order derivative of the position and refers to the rate at which the tibia rotates and translates over the femur with respect to squared time (Eager et al., 2016). When there is a higher jerk, more sudden acceleration exists. In contrast, a more smoothed acceleration exists when the jerk is low (Eager et al., 2016). Thus, a higher jerk during the pivot shift maneuver suggests a decrease in rotational knee stability (less smoothened motion), suggesting a non-physiological shift of the rotation knee axis (Domnick et al., 2016). *In vitro* studies have shown a correlation between a positive pivot shift sign and a deficiency of horizontal fibers of the ACL (Domnick et al., 2016). Moreover, the complete insufficiency of the horizontal fibers of the ACL causes increased anterior tibial translation and rotatory instability of the knee (Zantop et al., 2007). Also, the increased shifting of the rotation axis is a known factor to predispose the ACL rupture, allowing unstable rotational directions (Domnick et al., 2016). Thus, our findings in the pivot shift maneuver suggested that a non-physiological axis movement can occur without significant internal rotation during the dynamic landing task. As predicted, this finding was because of the dynamic pivoting landing task, which has shown more sensitivity to completing insufficient ACL (Pan et al., 2020). Instead, the dynamic testing increases the muscle activation to control the landing (Leporace et al., 2011). Therefore, the sensitivity to detect the axis movement alteration using the dynamic test following a competitive soccer match could be less than that with the pivot testing to find rotatory knee alterations in non-complete insufficient ACL.

The pivoting knee movements are expected to be controlled by knee co-contraction (Hirokawa et al., 1991; O’Connor, 1993) and by an adequate knee laxity (Bull and Amis, 1998). However, following a competitive soccer match, a deficient muscular action and/or muscle strength loss, the main factors that affect the knee joint stability (Kellis and Kouvelioti, 2009), may have allowed the increase of the pivoting motion of the knee in our study (increased acceleration of the pivot shift). Although knee laxity has been reported after a short period of no exhausting exercise, altered knee kinematics can arise when the exercise duration increases (Shultz et al., 2015), such as our study suggests. In particular, it is known that the fatigue measurements during competitive soccer matches elicit a decline in the maximal voluntary contraction (−14%), the decline of neuromuscular stimulation, a decrease in the jump performance, and a fatigue perception in the range of 24–72 h after the match (Brownstein et al., 2017). Our study has observed an acute pivoting knee adaptation where the fatigue induced by soccer playing appears to be the most relevant source of pivot shift alteration. As our results showed significant differences detected in the static pivot shift test but not in the dynamic pivoting landing task, the more knee muscle activation required during the landing of the pivoting task would help maintain the knee stability (Leporace et al., 2011), even if there was a decreased passive stability (laxity), such as the pivot shift maneuver suggests have occurred in our study.

Regarding the pivot shift acceleration for the preferred limb and the jerk for the preferred and non-preferred limbs following a competitive soccer match, this asymmetry could be related to the muscle strength asymmetries that characterize soccer players (Fousekis et al., 2010a). Soccer practices and competitions induce asymmetries. However, these adaptations tend to increase in non-professional players as well as players with intermediate and short training age (Fousekis et al., 2010b; Bishop et al., 2021). Thus, these asymmetries might have influenced the asymmetric results found in the pivot shift maneuver in our study.

We did not find statistical significance in the correlation between performance parameters and knee stability. However, the internal knee rotation had a higher inverse correlation with average heart rate, the distance at high-intensity speed, and maximum speed reached. In comparison, the jerk of pivot shift had a higher inverse correlation for total distance covered and the number of efforts at high intensities, and the acceleration of the pivot shift had a higher inverse correlation only with the total distance covered. These correlations suggest that whether the internal knee rotation, acceleration, and jerk of the pivot shift increase, the performance parameter, which was related with rapid maneuvers, decreases. These findings are relevant for athletes because they are more exposed to maneuvers at high speed, needing a high level of dynamic knee stability (Markström et al., 2019). Thus, the altered measurements of knee stability might suggest an immediate compromise of the performance parameters.

In conclusion, regarding the practical appraisal, coaches and medical teams must augment the surveillance of muscle fatigue in training and match sessions to effectively manage the induced risk of ACL and other injuries that the own soccer match can cause.

Our study is not without limitations. The most limiting assumption was the lack of direct fatigue measurement after the soccer match. This would have complemented our interpretations on the Borg scale better. Another limitation was the lack of neurophysiological measurements like direct measurements of muscle fatigue. We were not able to do it because it would have added an excessive assessment time. The sample size for the correlation may have been insufficient to find statistical differences and should be interpreted carefully.

## Conclusion

Rotatory stability decreases following a competitive soccer match in amateur soccer players under fatigue induced by soccer playing. Both the acceleration and jerk during the pivot shift maneuver are increased without significant internal knee rotation changes during the pivoting landing task suggesting a non-physiological shifting of the rotational knee axis and likely increased knee laxity.

## Data Availability

Data from the study is available in https://www.researchgate.net/publication/359410062_Is_the_rotatory_knee_stability_immediately_decreased_following_a_competitive_soccer_match_Brief_research_report.
